# Structure-Function-Immunogenicity Studies of PfEMP1 Domain DBL2β_PF11_0521_, a Malaria Parasite Ligand for ICAM-1

**DOI:** 10.1371/journal.pone.0061323

**Published:** 2013-04-12

**Authors:** Justin Gullingsrud, Tracy Saveria, Emily Amos, Patrick E. Duffy, Andrew V. Oleinikov

**Affiliations:** 1 Seattle Biomedical Research Institute, Seattle, Washington, United States of America; 2 Department of Global Health, Program of Pathobiology, University of Washington, Seattle, Washington, United States of America; 3 Laboratory of Malaria Immunology and Vaccinology, NIAID/NIH, Rockville, Maryland, United States of America; King Abdullah University of Science and Technology, Saudi Arabia

## Abstract

*Plasmodium falciparum* virulence has been ascribed to its ability to sequester in deep vascular beds, mediated by the variant surface antigen family PfEMP1 binding endothelial receptors like ICAM-1. We previously observed that naturally-acquired antibodies that block a PfEMP1 domain, DBL2β of PF11_0521 allele, from binding to the human ICAM1 receptor, reduce the risk of malaria hospitalization in children. Here, we find that DBL2β_PF11_0521_ binds ICAM-1 in the low nM range and relate the structure of this domain with its function and immunogenicity. We demonstrate that the interaction with ICAM-1 is not impaired by point mutations in the N-terminal subdomain or in the flexible Loop 4 of DBL2β_PF11_0521_, although both substructures were previously implicated in binding ICAM-1. These data will help to refine the existing model of DBLβ::ICAM-1 interactions. Antibodies raised against full-length DBL2β_PF11_0521_, but not truncated forms lacking the N terminal fragment, block its interaction with ICAM-1. Our data suggest that full length domain is optimal for displaying functional epitopes and has a broad surface of interaction with ICAM-1 that is not disrupted by individual amino acid substitutions at putative key residues. This information might be important for the future design of anti-malarial vaccines based on PfEMP1 antigens.

## Introduction

Cytoadherence plays an important role in the lifecycle and virulence *of P. falciparum*, the deadliest of human malaria parasites. The sequestered mass of adherent parasites leads to vascular occlusion that strongly correlates with disease severity [1] and inflammation in different organs. The main parasite ligand for infected erythrocyte (IE) adhesion is a family (∼60 members) of PfEMP1 proteins [2]–[4] that appear on the surface of IE in specialized structures called “knobs” [5]. Cytoadhesion of IE is a cause or feature of cerebral malaria (CM) [6]–[8] and of pregnancy malaria (PM) [9].

Each PfEMP1 protein consists of single intracellular and trans-membrane domains, and several extracellular domains (2 to 7 domains, ∼30–45 kDa per domain) heavily cross-linked by disulfide bonds. All PfEMP1 domains have been classified into several sub-classes: Duffy binding like (DBL) α, β (previously called βC2, a combination of the β and C2 domains), γ, δ, ζ, ε, and X; and cysteine-rich interdomain regions (CIDR) α, β, and γ [4], [10], [11]. DBL domains all have a similar scaffold [12]–[15]; the C-terminal part of the CIDR scaffold is similar to the C-terminal part of the DBL domain [16]. These domains are responsible for adhesion to various host receptors (reviewed in [17]). The host receptor binding specificity and combination of individual domains in the expressed PfEMP1 proteins may determine final IE adhesion specificity, strength of binding, and possibly, different pathologies related to malaria syndromes [18].

Various refinements of domain classification and of domain boundaries resulted from recent analyses of PfEMP1 sequences from 7 parasite genomes [19]. For example, in the previous terminology DBLβ and C2 were considered as separate domains that are found always together as a DBLβC2 combination. However, domain C2 is essentially a part of the same β domain [20], [21]. Therefore, DBLβ is a more correct term for this class of domains [19] which we will use henceforth.

Though its role remains inconclusive, ICAM-1 has been proposed as the key host adhesion receptor for iRBC causing CM [7], [8]. In support of this, parasites isolated from patients with CM bind at higher levels to ICAM-1 [22]. Domains of the DBLβ class have been implicated as ligands for ICAM-1 [10], although most DBLβ domains do not bind this host receptor. For example, only 6/24 DBLβ domains from the IT4 genome bound to ICAM1 *in vitro* [20]. In our previous work, only 1/18 DBLβ domain from the 3D7 genome, specifically the DBL2β domain of PF11_0521 allele, showed strong avidity binding to ICAM-1 [21]. We also recently observed that antibodies to DBL2β_PF11_0521_ predict reduced risk of hospitalization for malaria among Tanzanian children [23]. Therefore, understanding the molecular details of the interaction between ICAM-1 receptor and the DBL2β domains, and the structural features leading to a functional immune response against these domains might guide the design of anti-severe malaria vaccines and drugs.

Interactions between ICAM-1 and the DBL2β domain were previously modeled *in silico*, based on several lines of evidence, such as the key role of a single amino acid residue in loop 4 of the DBL2β domain [24]. We have subsequently shown [21] that DBL2β domains are about 70 amino acid residues longer than previously assumed [10], and that this extra-N-terminal fragment (not included in the *in silico* model) is important for functional activity [21]. Here, we use site-specific mutagenesis of residues in putative key sub-structures of the DBL2β_PF11_0521_ domain to assess their roles in binding ICAM-1. We also examined the functional anti-adhesion activity of antibodies raised against truncated and full-length variants of DBL2β_PF11_0521_ to better understand the requirements for PfEMP1 domain-based vaccines that might prevent iRBC adhesion and thus severe malaria.

## Materials and Methods

### Ethics statement

Animal ethics adhered to specific national and international guidelines. Animal use protocols “Antibodies Inc. Protocol (Offsite)” and “Liver Stage Vaccines” meet the standards of the *Guide for the Care and Use of Laboratory Animals* (by National Academy of Sciences) and applicable Seattle Biomedical Research Institute (Seattle BioMed) policies and procedures. Seattle BioMed has an Animal Welfare Assurance (A36640-01) on file with the NIH Office of Laboratory Animal Welfare. Protocols #AO-06-ABP and #AO-02 have been approved by Seattle BioMed IACUC committee. All animals used in the experiments were observed on a daily routine for presence or absence of distress and/or signs of illness, timely veterinary care was provided as needed. Euthanasia method via exsanguination by cardiac puncture under ketamine/xylazine anesthesia was used.

### DBL2β_PF11_0521_ constructs

**Figure 1:**
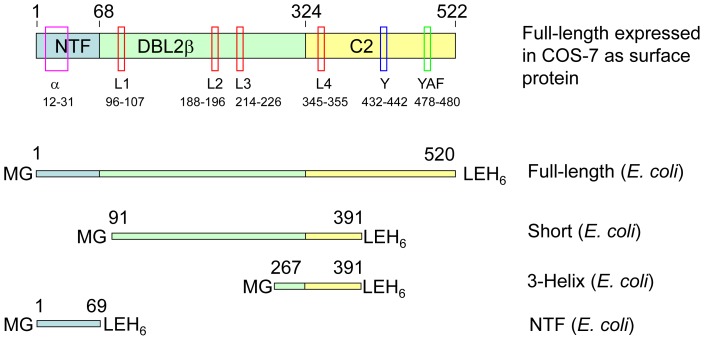
Schematic representation of several DBL2β domain structural features and constructs expressed in *E. coli*. For relevance to older works, domain is shown as a combination of NTF, DBLβ and C2 parts according to previous nomenclature. NTF, N-terminal fragment. Semi-conserved and conserved features [20], [21], [24] are indicated by black boxes: α, first alpha helix; L1 through L4, Loops 1 through 4; Y, Y – motif; YAF, Tyr-Ala-Phe. Numbers above proteins and under structural features indicate position of amino acid (AA) residues. Additional AA residues at the protein ends, which appear as a result of cloning, are indicated for each *E. coli* expressed fragment using single letter code.

Cloning of the full-length DBL2β_PF11_0521_ domain and its expression in COS-7 cells as a surface-expressed molecule was described in [21]. Figure 1 schematically shows full-length DBL2β_PF11_0521_ domain expressed in COS-7 cells and several domain structural features, as well as constructs (expressed in *E. coli*) used in this work. The nucleotide and corresponding protein sequences for this domain, the various truncation and point-mutation constructs, as well as names of relevant cloning vectors are all shown in Figure S1. The following amino acid (AA) substitutions were made using Quick Change mutagenesis [25] with Pfu turbo polymerase (Stratagene) in the full-length domain that was cloned into pHisAdEx vector [21] for expression in COS-7 cells: A25K; R23A+A25K; A347L; A347H; A347Y.

Full-length DBL2β domain (AA 1 through 520 corresponding to AA 728–1247 in the sequence of full-length PF11_0521 PfEMP1 protein, starting from its initiator methionine) and its truncated fragments AA 91–391 (“short”), AA 267–391 (“3-helix”), and the N-terminal sub-domain (N-terminal fragment, NTF, AA 1–69) were cloned into the pET28b vector, expressed in *E. coli*, purified by Ni-column followed by Reverse Phase HPLC and refolded as described earlier [26]. These proteins were assessed for binding to ICAM-1 and used for preparation of antibodies.

We expressed HisAdEx [21] without additional insert in COS-7, and AMA-1 [27] in *E. coli* using pET28b with subsequent purification and re-folding by the same methods described above for full-length DBL2β_PF11_0521_ domain.

### DBL2β_PF11_0521_ and ICAM-1 binding and binding-inhibition assays

We performed binding and binding-inhibition studies as previously described, using BioPlex (BioRad) approach, COS-7 expressed proteins, and ICAM-1-Fc chimera (R&D Systems) [21]. All measurements were made in duplicate. In each experiment, the amount of recombinant domain immobilized on the beads was measured by reactivity with antibodies to full length *E.coli*-expressed/refolded domain (see below), and this amount then used to normalize ICAM-1-binding levels [27]. *E. coli*-expressed and purified domains were immobilized on carboxy-modified polystyrene beads (BioRad) using the manufacturer-supplied protocol and assayed in the same fashion as bead-immobilized COS-7-expressed proteins. COS7-expressed GFP-fused proteins were immobilized on the beads through capture by anti-GFP antibodies previously immobilized by chemical cross-linking [21] and E. coli-expressed domains through direct random cross-linking. For comparison of specific binding activity of *E. coli* and COS-7 expressed proteins, the amount of protein bound to the beads was measured by reactivity with anti-full length *DBL2β_PF11_0521_* domain sera (rat sera to *E.coli*-expressed domain) as described below.

### Preparation of antibodies against domain variants

Purified and refolded *E. coli*-expressed domain variants were used for preparation of antibodies in rats at Antibodies, Inc. (Davis, CA) using their standard schedule protocol and 10 µg of antigen per injection, and in mice at Seattle BioMed as described earlier [26]. DNA immunization using full-length domain cloned into pHisAdEx was performed at the Seattle BioMed animal facility as follows: female Balb/c mice aged 6–12 weeks received 4 immunizations (∼50 µg DNA) at 3-week intervals intramuscularly. Terminal bleeds were collected 10–14 days after the final immunization. Animal work was described in protocols listed in *Ethical Statement* above and has been approved by IACUC at Seattle BioMed.

### Reactivity of anti-domain antibodies with various DBLβ_PF11_0521_ domain constructs

A range of dilutions of anti-sera raised against various domain constructs was incubated with different bead-immobilized domain constructs for 1 hour at room temperature (RT). Pooled pre-immune sera and anti-sera from animals raised against control proteins (HisAdEx or AMA1) were used as negative controls. We used pooled sera from all animals immunized with the same antigen. Beads were washed 3 times with phosphate buffered saline (PBS)-0.05% Tween-20 (PBST), incubated with goat anti-species IgG coupled to PE (Jackson Immunoresearch, dilutions: 1∶5000 anti-rat, 1∶1000 or 1∶500 anti-mouse) for 1 hour at RT, washed 3 times in PBST and 1 time with PBS, and finally re-suspended in 125 µl of PBS. Signals were measured on BioPlex 200 (BioRad) as described in our publications [21], [23].

### Determination of avidity constant for binding of DBL2β_PF11_0521_ and ICAM1

We used full-length DBL2β domain expressed in both systems (COS-7 and *E. coli*) and immobilized on BioPlex beads (as described above) for these measurements. *E. coli*-expressed protein was refolded as described above. Initial velocity of binding was measured at various concentrations (0.3, 1, and 3 ug/ml) of ICAM-1-Fc. At these concentrations and incubation times (5 to 35 min), the level of ICAM-1 binding to bead-immobilized domain was less than 10% of the maximum. The incubations and measurements were performed in a homogenous system; ICAM-1-Fc was pre-incubated for 1 hour with an excess of donkey anti-human IgG coupled to phycoerythrin (PE) detection molecules, and then added to beads with immobilized full-length DBL2β domain. Final concentrations of ICAM-1 in these mixtures are shown above and the final dilution of anti-human IgG-PE was 1∶250. After incubation for the indicated periods of time, beads were subjected to signal measurements using a Bio-Plex 200 machine (BioRad) without any washing steps. The actual time (in seconds) between mixing ICAM-1-Fc and donkey anti-human IgG-PE with beads and signal measurement events on the machine was used for kinetics calculations. As active ICAM-1-Fc molecules are organized as dimers, we used its molecular mass equal to ∼200 kDa (R&D Systems) for calculations. We used the Michaelis-Menten equation and Lineweaver-Burk plots [28] for the calculation of the equilibrium dissociation constant K_D_. Linear regression lines and 95% confidence intervals (CI) were calculated using GraphPad Prizm software.

*Visualization of DBL2β::ICAM-1 interacting molecules and* in silico *amino acid substitution analyses* were performed using functions of Deep View Swiss-PDB viewer and Bertonati and Tramontano model [24].

*Secondary structure prediction* was performed using consensus prediction [29] at Pôle Bioinformatique Lyonnais web site (http://www.ibcp.fr/predict.html).

## Results and Discussion

A model of *DBL2β::ICAM-1* interactions suggested by Bertonati and Tramontano [24] was obtained by computational docking of the X-ray resolved 3-D structure of ICAM-1 N-terminal domain and the predicted 3-D structure of PfEMP1 DBL2β domain, in turn modeled using the available X-ray resolved 3-D structure of the homologous F1 domain in EBA 175 malarial protein [13], [30]. This model explains or is in agreement with several experimental observations regarding PfEMP1 domain::ICAM-1 interactions (discussed in [24]) and may serve as a basis for design of various biochemical experiments for further characterization of DBL2βICAM-1 domain interactions.

However, the Bertonati-Tramontano model has limitations, and several observations cannot be explained by this model. For example, the Y-motif in the C2 part of the domain (Figure 1 and Figure S2A) was shown to be critical for ICAM-1 binding [31], and chimeric constructs indicate interdependence between the C2 and the upstream components of the domain [31]. Despite these experimental data, the Y motif of C2 is distant from the ICAM-1 interaction site in the model of Bertonati and Tramontano, and does not make contacts with ICAM-1, nor substantial contacts with the rest of the DBLβ domain (Figure S2A). In addition, a completely conserved Tyr-Ala-Phe (YAF) motif located in the C2 part of the DBLβ domain (Figure 1) is not incorporated in this model. The importance of the C-terminal third of the DBLβ domain from another PfEMP1 protein, PFD1235w, for ICAM-1 binding was confirmed recently [32] but the implicated part (∼180 AA) includes sequences that are involved in the interactions according to the model as well as those that are distant.

Different parasite strains [21], [24], [31] consistently have A, L, or H residues in position 286 within flexible loop 4 (AA number according to [24], in our full-length domain this is number 347) of ICAM-1 binding parasites, while non-binding strains contain E, K, Q, W, S, Y as well as A, L, and H. E and K are the most common residues in this position. The Bertonati-Tramontano model predicts that several residues including AA286 in the DBLβdomain make close contact with ICAM-1 (Figure S2). However, *in silico* substitutions of amino acid residue 286 in the model [24] that we performed using Deep View Swiss-PDB viewer indicate that even those residues that are present in Loop 4 in ICAM-1 non-binding strains (including E and K) are sterically compatible with the modeled interactions, and may be energetically advantageous (Figure S2 B and C). Therefore, we tested *in vitro* the effect of substitutions of amino acid residue 286 on DBL2β::ICAM-1 interactions.

Finally, we have shown [21] that the previously defined DBLβ domain [10] used for modeling [24] was missing an additional N-terminal subdomain. This has subsequently been confirmed in analyses of seven *P. falciparum* genomes [19]. Moreover, for the 3D7 DBL2β_PF11_0521_ domain, which strongly and specifically binds ICAM-1, we reported that the N-terminal subdomain contributes to ICAM-1 binding activity [21]. Therefore, we also made point mutations in a conserved alpha-helix (Figure S3) predicted by consensus secondary-structure analysis [29] to be a prominent structural element in this N-terminal subdomain [21]. As we wanted to preserve the structure of this alpha-helix but disturb possible interactions, we made substitutions that significantly changed the physical-chemical nature of the amino acid residues but did not affect the formation of this α-helix. Secondary structure analysis of our point mutation sequence variants according to Deleage et al. [29] predicts preservation of this α-helix.

**Figure 2:**
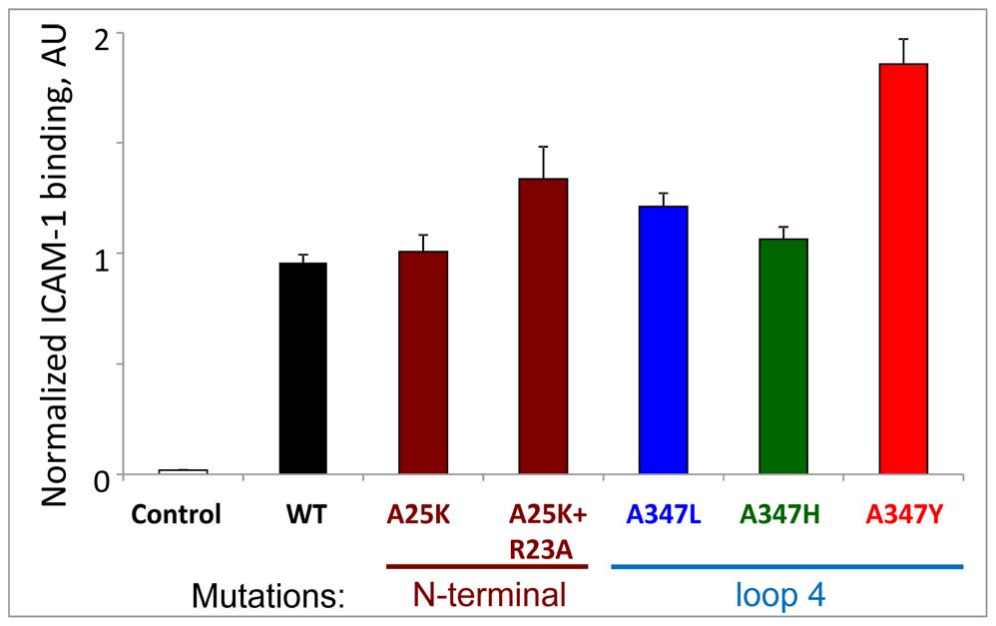
ICAM-1 binding by mutated DBL2β constructs. Single or double mutations in N-terminal sub-domain or single mutations (A→L, H, Y) in Loop 4 do not reduce ICAM-1 binding activity. WT, wild type. AU, arbitrary units. Error bars represent Standard Deviations. Control, HisAdEx negative control construct.

As expected for two proteins with substantial surface area contacts, none of the single point mutations in the DBLβ domain diminished binding interactions with ICAM-1 (Figure 2), presumably because they did not disrupt overall folding of the protein. Preservation of overall folding would be expected for the substitutions made in flexible loops. Similar results were obtained in previous work in which Alanine replacements of at least 3 residues (different from those reported here) were required to reduce binding interactions [20]. In our mutation experiments, the substitution of a natural Ala286 residue in Loop 4 (number 347 in our full-length construct) to any of the tested residues did not reduce interactions. Moreover, substitution with Tyr, the residue that occurs only in ICAM-1 non-binding variants, yielded an even stronger interaction with ICAM-1, in agreement with our *in silico* modeling of Tyr substitution (above) that predicts an additional intra-molecular hydrogen bond (between Tyr 186 and Arg113 of the domain) that may stabilize the complex (Figure S2 B and C). However, this agreement with the Bertonati and Tramontano model does not explain the observation that only A, L, and rarely H residues appear at this position in ICAM-1 binding DBLβ domain variants. More DBLβ domain variants should be tested in order to strengthen or refute the association of A, L and H residues at this position, with binding to ICAM-1.

Similar to the Loop 4 findings, substitutions in the N-terminal subdomain α-helix did not affect ICAM-1 binding (Figure 2). Thus, these amino acid residues are not crucial for interactions with ICAM-1. These data further support the notion that the two molecules have substantial surface interactions that are not affected significantly by point mutations that preserve overall structure.

We have expressed four structural variants of DBL2β_PF11_0521_ domain (Figures 1 and S1) in *E. coli*, then purified and refolded (at least made soluble) these: the full length domain (sequence as described in [21]); a truncated construct designed according to the minimal binding fragment (MBF) [31] and called “short”; a construct called “3-helix” that comprises the three helices involved in forming Loop 4 (Figure S2), which interacts with ICAM-1 according to the model [24]; and N-terminal subdomain (also called here as N-terminal fragment, NTF). Of note, the proposed MBF does not bind ICAM-1 [31]: although a series of sequential DBLβdomain truncations performed independently at the N- and C-termini suggested this fragment to be the MBF, it is not functional as a single construct.

**Figure 3:**
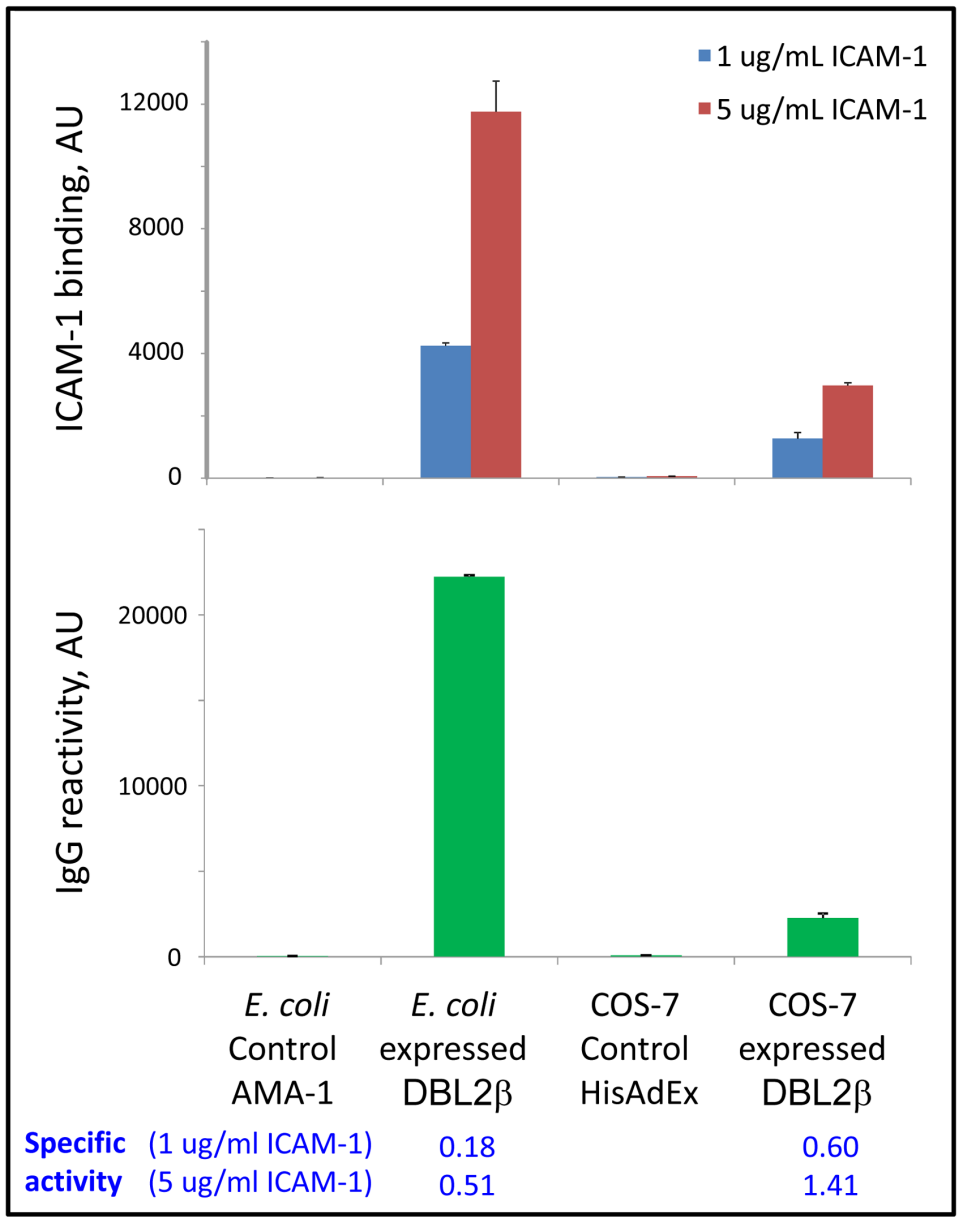
*E. coli* expressed/refolded PF11_0521 DBL2 domain binds ICAM-1 but has ∼3 times lower specific activity than COS-7 expressed domain. Amount of protein on beads was measured by reactivity of rat plasma IgG raised against *E. coli*-expressed full-length DBL2 domain (in arbitrary units, AU). Error bars represent Standard Deviations. Dilution used (1∶32000) is in the linear range of titration curve for these antibodies. Specific activity = AU(ICAM-1 binding)/AU(IgG reactivity).

In accordance with our previous results using COS-expressed truncated domains [21], the “short” and “3-helix” DBL2β_PF11_0521_ domain variants did not bind ICAM-1 (data not shown), most likely due to incorrect folding of these truncated domain fragments (see below). Refolded full-length recombinant domain strongly bound the ICAM-1 receptor (Figure 3), though the specific activity (ICAM-1 binding per amount of domain immobilized on the bead as measured using antibody to *E. coli*-expressed full length domain, described below) was ∼3–fold lower than that seen with COS-7-expressed protein. This indicates that only a fraction of the *E. coli* preparation (∼33%) is refolded correctly to the active conformation or is sufficiently stable for binding on the beads. Random immobilization (of the *E. coli*-expressed protein) may inactivate some number of molecules by their unfavorable cross-linking on the surface of the beads. This may contribute to the inactive pool of bead-immobilized domain molecules and indicates that at least 33% of the refolded soluble preparation was active before cross-linking. Directed immobilization (of the COS-expressed domain) in this respect is preferable over the random immobilization.

Binding between the DBL2β_PF11_0521_ domain and ICAM-1 receptor is strong, with a calculated equilibrium dissociation constant (K_D_) in the low nM range (2.3–2.6 nM; 95% CI = 1.3–4.4 nM) (Figure S4). The high avidity receptor binding is in accordance with the notion that parasite cytoadherence may continue for many hours after killing parasites with conventional drugs, contributing to pathophysiology of severe disease [33], and with the presumptive role for ICAM-1 receptor in severe malaria [10], [22]. The strong avidity constant for ICAM-1 binding also supports previous results on the important role for ICAM-1 receptor in tight binding of infected erythrocytes to endothelial cells under flow [34]. High avidity binding can also explain why naturally acquired antibodies fail to reverse this interaction [21], [35] even though they efficiently block it [21]. Most adults but not children living in malaria endemic regions have acquired antibodies that block binding to ICAM1 [21], [23], and these might protect against severe malaria by limiting new parasites from sequestering, even though binding is difficult to reverse once adhesion has occurred.

We have previously demonstrated that antibodies against DBL2β_PF11_0521_ domain are common in malaria-immune adults [21], appear early in life, and are associated with protection against severe disease in young children [23]. Therefore, we were interested to determine whether antibodies raised against the recombinant DBL2β_PF11_0521_ domain would affect binding of this domain to ICAM-1, and as well to determine the structure and size of the domain needed to re-create functional epitopes. All four *E. coli*-expressed DBL2β_PF11_0521_ domain constructs (full-length, “short”, “3-helix”, and NTF) were used for immunizations of rodents, as was plasmid DNA pHisAdEx-DBL2β_PF11_0521_ containing full length domain. This latter construct was previously used for production of functionally active protein in COS-7 cells [21] and is the base construct for mutagenesis and ICAM-1 binding experiments in this work.

**Figure 4:**
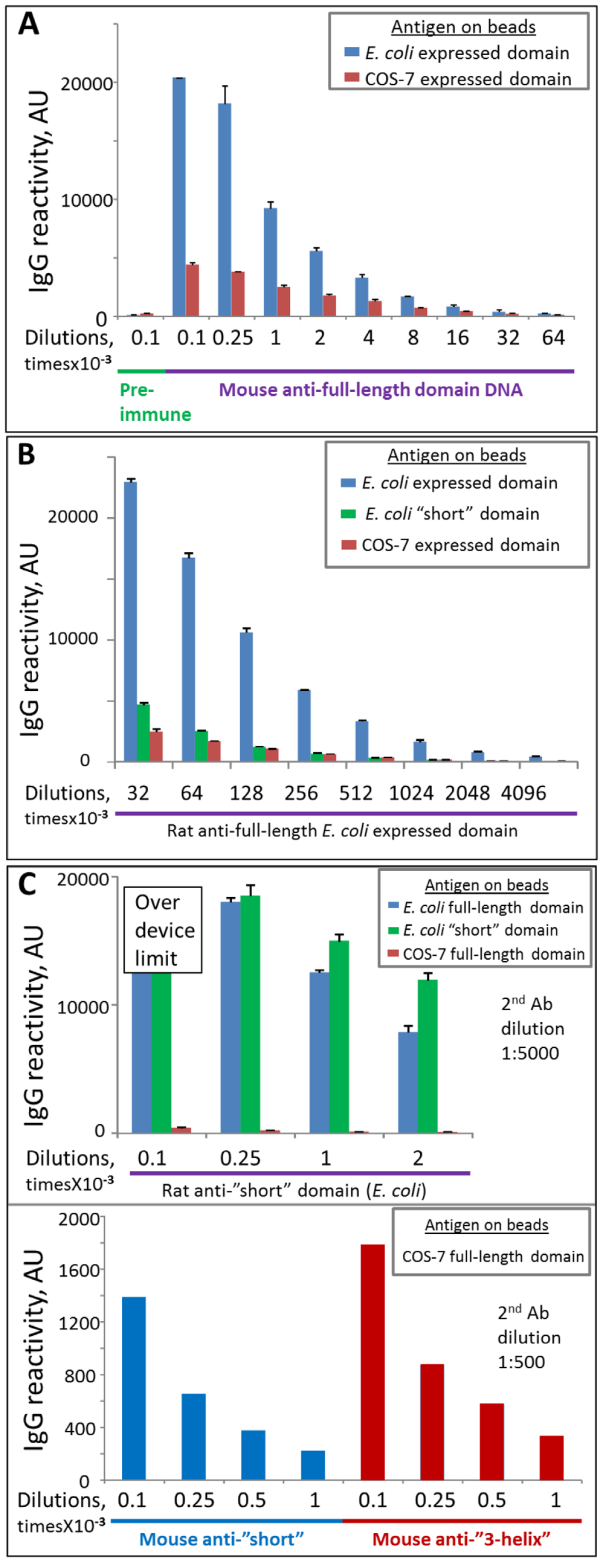
Reactivity of various antibodies to bead-immobilized COS-7 and *E. coli*-expressed domain variants. **A**. Mouse antibodies to full-length domain prepared by DNA immunization. No reactivity to “short” domain was observed (data not shown). **B**. Rat antibodies to *E. coli*-expressed full–length domain. Error bars represent Standard Deviations. **C**. Rat (top) and mouse (bottom) antibodies to *E. coli*-expressed “short” and “3-helix” domains.

Mouse anti-full-length domain antibodies prepared by DNA immunization recognized both *E. coli* and COS-7 expressed full-length domains (Figure 4A), with similar endpoint titers down to 1∶64,000. The level of reactivity was higher for the beads displaying the *E. coli*-expressed domain because more protein bound to these beads, compared to beads displaying COS-7 expressed protein (as was found using antibodies raised against *E. coli*-expressed domain, see below). Antisera collected after DNA immunization failed to react to the “short” E. coli expressed truncated domain, which represents a substantial portion of the full-length protein but, as we suggested, is mis- or un-folded. We conclude that intramuscular DNA immunization yielded correctly folded domain on the surface of the host cells and induced antibodies that predominantly recognize surface-exposed structural epitopes of this correctly folded form, whereas antibodies raised against *E. coli*-expressed protein (see below) recognize epitopes of both folded and unfolded protein and might therefore reflect total protein in a preparation rather than solely the binding form of protein. Because antibodies raised by DNA immunization recognize only the folded form of the domain, they cannot be used to determine the amount of total protein immobilized on the beads.

Rat antisera against *E. coli* expressed/refolded full-length domain recognized both *E. coli* expressed/refolded full length domain and COS-7 expressed domain (Figure 4B), with titers down to 1∶4,096,000. These antisera also strongly reacted with inactive “short” mis/unfolded domain (Figure 4B), presumably because a fraction of the *E. coli* product used for immunization included mis- or un-folded protein. Using these antisera, we quantified the amount of protein on the BioPlex beads, and then estimated the specific (normalized) binding activity of mutant variants, and compared specific ICAM-1 binding activity of *E. coli* expressed/refolded and COS-7 expressed protein (above).

**Figure 5:**
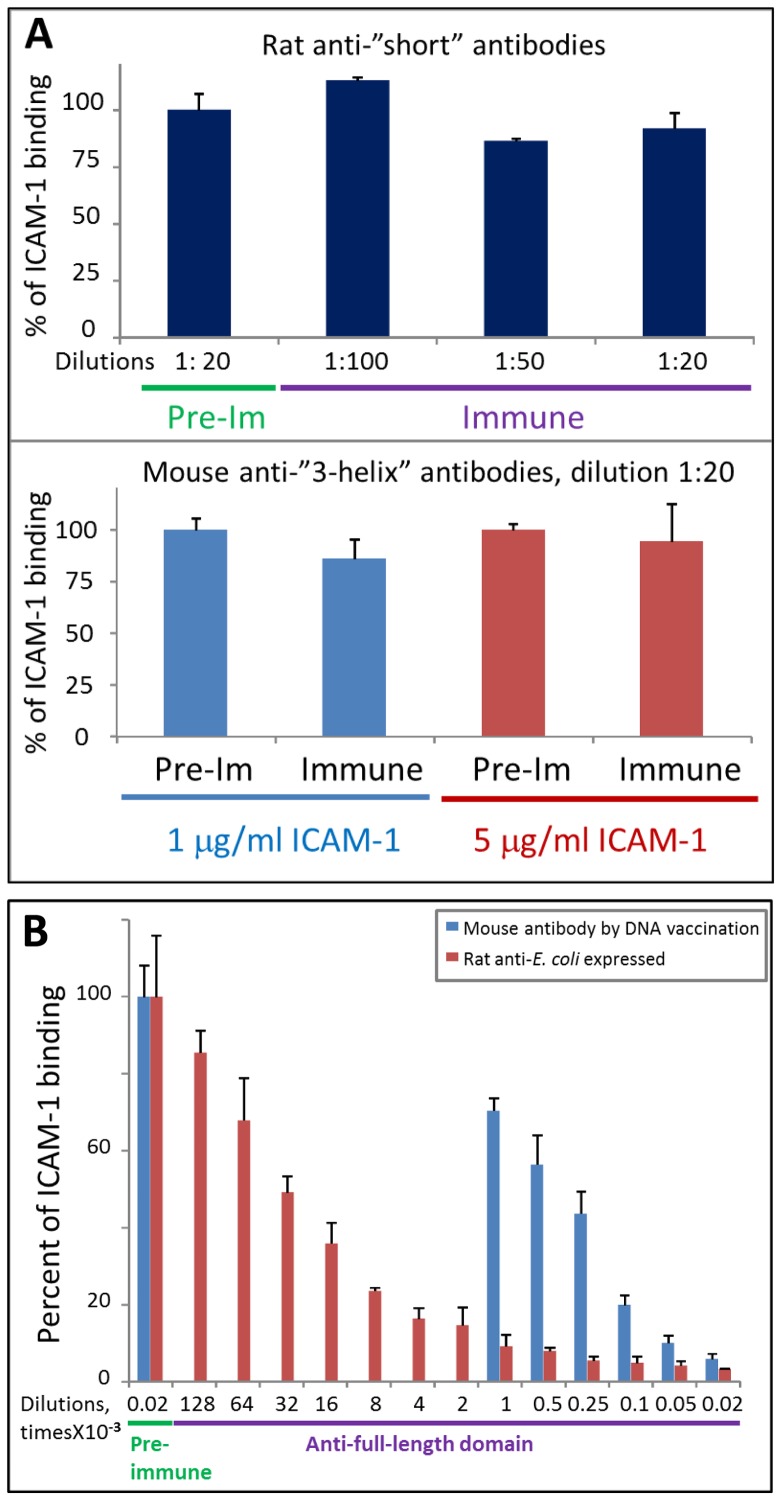
Functional activity of animal antibodies against various DBL2 domain constructs. **A**. Antibodies against “short” (top) and “3-helix” (bottom) domains do not inhibit ICAM-1 binding to COS-expressed full-length domain. Pre-Im, pre-immune plasma as negative control. Error bars represent Standard Deviations. **B**. Antibodies against full-length domain can completely inhibit ICAM-1 binding to COS-expressed full-length domain. Mouse antibodies were titrated down to 1∶1000 dilution.

Rat anti-“short” antibodies strongly and comparably recognized full-length and “short” *E. coli* expressed domains but recognition of COS-7 expressed full-length domain was significantly weaker (∼12 times less reactivity normalized to the amount of protein on beads) (Figure 4C, top), again indicating that the majority of the COS-7 expressed protein is folded and that cryptic or linear epitopes, the main targets of “short” domain antigen, are not accessible. However, the recognition is clearly detectable using a lower dilution of secondary antibodies (exemplified in Figure 4C, bottom, using antibodies raised in mice), indicating that these antibodies either recognize some small number of epitopes (most likely linear) in the full-length domain, or the recognition is of low affinity, or the COS-7 expressed preparation also contains some small amount of mis- or un-folded protein (or a combination of all three). These antibodies did not inhibit binding of the COS-expressed domain to ICAM-1 (Figure 5A). Similar behavior was observed for antibodies prepared in rats and mice. Mouse anti-“3-helix” antibodies behaved similarly and did not inhibit ICAM-1 binding (Figure 5A) but had slightly higher reactivity than anti-“short” antibodies (Figure 4C, bottom). Finally, rat anti-NTF antibodies behaved the same way as antibodies to both N-terminally truncated fragments (“short” and “3-helix”): they strongly recognized *E. coli*-expressed NTF and full-length domain but poorly recognized COS-expressed full-length domain and did not inhibit ICAM-1 binding (data not shown).

Antibodies raised against full-length domain (using DNA immunization or immunization with refolded *E. coli* expressed protein) were functionally active as they can completely inhibit ICAM-1 binding to COS-expressed DBL2β domain (Figure 5B). The titer of the antibodies obtained by DNA immunization was significantly lower than that obtained by protein immunization; the inhibitory activity was proportionally lower.

In summary, our results indicate that the prevailing model of DBL2β::ICAM-1domain interactions [24] is incomplete and may require further refinement to which our data will contribute. We cannot exclude though the possibility that variant DBLβ domains may interact differently with the ICAM-1 receptor and future studies on structure-functional relationships for other ICAM1-binding PfEMP1 domains (including experimental crystallographic data) will contribute to a better understanding of the precise molecular mechanisms of interactions. In our studies the Ala286Tyr (numbering according to [24]) amino acid residue substitution that has currently been found only in ICAM-1 non-binding domains does not reduce binding of DBL2β_PF11_0521_ domain. Point mutations in a predicted conserved α-helix located in the N-terminal sub-domain were also neutral. Our experiments with anti-DBL2β_PF11_0521_ domain antibodies (summary shown in Table S1) demonstrate that only antibodies to the full-length domain, but not to truncated fragments missing the N-terminal sub-domain or to N-terminal sub-domain, can inhibit interaction with ICAM-1. Our data indicate that truncated variants are likely mis- or un-folded. In addition, the full-length DBL2β_PF11_0521_ domain binds the ICAM-1 receptor with high avidity in the low nM range. These data indicate that selection of the correct boundaries of PfEMP1 domains is extremely important in the design of immunogens that will elicit functional adhesion-inhibitory antibodies.

## Supporting Information

Figure S1 **The nucleotide sequence for full-length DBL2β_PF11_0521_ domain and corresponding protein sequences for the various truncation and point-mutation constructs in various vectors.**
(DOCX)

Figure S2 **Structural elements of interacting ICAM-1 and DBLβ in the model of Bertonati and Tramontano.** Model [24] was visualized (**A**) and amino acid substitution in loop 4 (**B**) was modeled using Deep View Swiss-PDB viewer. **In panel A** loops 1 through 4 participating in interactions of DBLβ domain with ICAM-1 are shown in red, the rest of the molecule in green, ICAM-1 molecule in yellow. Ala286 residue in loop 4 that is involved in contacts with ICAM-1 is shown in blue. Y motif is circled. **In panel B** amino acid residue 286 (Ala or Tyr) is shown in pink. Amino acid residues Asn (N) and Tyr (Y), which are located at the beginning and the end of the loop 4, are indicated. Residues in the vicinity of the position 286 are shown in space-filling shape with CPK coloring. Substitution Ala286Tyr creates an additional hydrogen bond (**arrowhead in panel C**) between Tyr286 and Arg113 (numbers according to [24]).
(PPTX)

Figure S3 **Predicted secondary structure of N-terminal sub-domain and amino acid residue mutations in the first α-helix of DBL2β_PF11_0521_.** ICAM-1 binding and non-binding domains [20], [21] are grouped. Color of amino acid residues: purple – conserved at least in ICAM-1 binding sequences; blue – semi-conserved; red – having significantly different physical-chemical character from the majority of amino acid residues in this position that may affect structure or/and function of the domain. Site-directed mutations: R23→A, A25→K.
(PPTX)

Figure S4 **Determination of equilibrium dissociation constant K_D_ for ICAM-1 binding to PF11_0521 DBL2 domain.** Kinetics of binding for both *E. coli* expressed/refolded (ECO, N = 18) and COS-7 expressed (COS, N = 14) domains was measured in 4 independent experiments. 95% confidence intervals (CI) shown by dashed lines. Error bars are standard errors of mean. N, number of replicates for each concentration point. Formulas show linear regressions. V, initial velocity of binding; C, concentration of ICAM-1; min, minutes, AU, arbitrary units.
(PPTX)

Table S1 **Summary of results of binding and inhibition of binding of ICAM-1 receptor obtained with various constructs of DBLβ_PF11_0521_ and antibodies against these constructs.**
(DOCX)

## References

[1] Dondorp AM (2008) Clinical significance of sequestration in adults with severe malaria. Transfus Clin Biol 15: 56–57 Epub 2008 May 2023. 18501654 10.1016/j.tracli.2008.04.013

[2] Su XZ, Heatwole VM, Wertheimer SP, Guinet F, Herrfeldt JA, et al (1995) The large diverse gene family var encodes proteins involved in cytoadherence and antigenic variation of Plasmodium falciparum-infected erythrocytes. Cell 82: 89–100. 7606788 10.1016/0092-8674(95)90055-1

[3] Smith JD, Chitnis CE, Craig AG, Roberts DJ, Hudson-Taylor DE, et al (1995) Switches in expression of Plasmodium falciparum var genes correlate with changes in antigenic and cytoadherent phenotypes of infected erythrocytes. Cell 82: 101–110. 7606775 10.1016/0092-8674(95)90056-x PMC3730239

[4] Gardner MJ, Hall N, Fung E, White O, Berriman M, et al (2002) Genome sequence of the human malaria parasite Plasmodium falciparum. Nature 419: 498–511. 12368864 10.1038/nature01097 PMC3836256

[5] Biggs BA, Gooze L, Wycherley K, Wilkinson D, Boyd AW, et al (1990) Knob-independent cytoadherence of Plasmodium falciparum to the leukocyte differentiation antigen CD36. J Exp Med 171: 1883–1892. 1693652 10.1084/jem.171.6.1883 PMC2187967

[6] Miller LH, Good MF, Milon G (1994) Malaria pathogenesis. Science 264: 1878–1883. 8009217 10.1126/science.8009217

[7] Newbold C, Warn P, Black G, Berendt A, Craig A, et al (1997) Receptor-specific adhesion and clinical disease in Plasmodium falciparum. Am J Trop Med Hyg 57: 389–398. 9347951 10.4269/ajtmh.1997.57.389

[8] Turner GD, Morrison H, Jones M, Davis TM, Looareesuwan S, et al (1994) An immunohistochemical study of the pathology of fatal malaria. Evidence for widespread endothelial activation and a potential role for intercellular adhesion molecule-1 in cerebral sequestration. Am J Pathol 145: 1057–1069. 7526692 PMC1887431

[9] Fried M, Duffy PE (1996) Adherence of Plasmodium falciparum to chondroitin sulfate A in the human placenta. Science 272: 1502–1504. 8633247 10.1126/science.272.5267.1502

[10] Smith JD, Craig AG, Kriek N, Hudson-Taylor D, Kyes S, et al (2000) Identification of a Plasmodium falciparum intercellular adhesion molecule-1 binding domain: a parasite adhesion trait implicated in cerebral malaria. Proc Natl Acad Sci U S A 97: 1766–1771. 10677532 10.1073/pnas.040545897 PMC26510

[11] Smith JD, Subramanian G, Gamain B, Baruch DI, Miller LH (2000) Classification of adhesive domains in the Plasmodium falciparum erythrocyte membrane protein 1 family. Mol Biochem Parasitol 110: 293–310. 11071284 10.1016/s0166-6851(00)00279-6

[12] McHenry AM, Adams JH (2006) The crystal structure of P. knowlesi DBPalpha DBL domain and its implications for immune evasion. Trends Biochem Sci 31: 487–491 Epub 2006 Jul 2027. 16876418 10.1016/j.tibs.2006.07.003 PMC2771397

[13] Singh SK, Hora R, Belrhali H, Chitnis CE, Sharma A (2006) Structural basis for Duffy recognition by the malaria parasite Duffy-binding-like domain. Nature 439: 741–744. 16372020 10.1038/nature04443

[14] Higgins MK (2008) The structure of a chondroitin sulfate-binding domain important in placental malaria. J Biol Chem 283: 21842–21846. 18550531 10.1074/jbc.C800086200 PMC2494935

[15] Singh K, Gittis AG, Nguyen P, Gowda DC, Miller LH, et al (2008) Structure of the DBL3x domain of pregnancy-associated malaria protein VAR2CSA complexed with chondroitin sulfate A. Nat Struct Mol Biol 24: 24. 10.1038/nsmb.1479 PMC2658892 19172746

[16] Klein MM, Gittis AG, Su HP, Makobongo MO, Moore JM, et al (2008) The cysteine-rich interdomain region from the highly variable plasmodium falciparum erythrocyte membrane protein-1 exhibits a conserved structure. PLoS Pathog 4: e1000147. 18773118 10.1371/journal.ppat.1000147 PMC2518858

[17] Howell DP, Samudrala R, Smith JD (2006) Disguising itself–insights into Plasmodium falciparum binding and immune evasion from the DBL crystal structure. Mol Biochem Parasitol 148: 1–9 Epub 2006 Apr 2004. 16621067 10.1016/j.molbiopara.2006.03.004

[18] Chakravorty SJ, Hughes KR, Craig AG (2008) Host response to cytoadherence in Plasmodium falciparum. Biochem Soc Trans 36: 221–228. 18363564 10.1042/BST0360221

[19] Rask TS, Hansen DA, Theander TG, Gorm Pedersen A, Lavstsen T (2010) Plasmodium falciparum erythrocyte membrane protein 1 diversity in seven genomes–divide and conquer. PLoS Comp Biol 6 (9) e1000933. 10.1371/journal.pcbi.1000933 PMC2940729 20862303

[20] Howell DP, Levin EA, Springer AL, Kraemer SM, Phippard DJ, et al (2008) Mapping a common interaction site used by Plasmodium falciparum Duffy binding-like domains to bind diverse host receptors. Mol Microbiol 67: 78–87. 18047571 10.1111/j.1365-2958.2007.06019.x

[21] Oleinikov AV, Amos E, Frye IT, Rossnagle E, Mutabingwa TK, et al (2009) High throughput functional assays of the variant antigen PfEMP1 reveal a single domain in the 3D7 Plasmodium falciparum genome that binds ICAM1 with high affinity and is targeted by naturally acquired neutralizing antibodies. PLoS Pathog 5: e1000386 Epub 1002009 Apr 1000317. 19381252 10.1371/journal.ppat.1000386 PMC2663049

[22] Ochola LB, Siddondo BR, Ocholla H, Nkya S, Kimani EN, et al (2011) Specific receptor usage in Plasmodium falciparum cytoadherence is associated with disease outcome. PLoS ONE 6: e14741. 21390226 10.1371/journal.pone.0014741 PMC3048392

[23] Oleinikov AV, Voronkova VV, Frye IT, Amos E, Morrison R, et al (2012) A Plasma Survey Using 38 PfEMP1 Domains Reveals Frequent Recognition of the *Plasmodium falciparum* Antigen VAR2CSA among Young Tanzanian Children. PLoS ONE 7: e31011. 22295123 10.1371/journal.pone.0031011 PMC3266279

[24] Bertonati C, Tramontano A (2007) A model of the complex between the PfEMP1 malaria protein and the human ICAM-1 receptor. Proteins 69: 215–222. 17640071 10.1002/prot.21691

[25] Wang W, Malcolm BA (1999) Two-stage PCR protocol allowing introduction of multiple mutations, deletions and insertions using QuikChange Site-Directed Mutagenesis. Biotechniques 10.2144/99264st03 10343905

[26] Oleinikov AV, Francis SE, Dorfman JR, Rossnagle E, Balcaitis S, et al (2008) VAR2CSA domains expressed in E.coli induce cross-reactive antibodies to native protein. Journal of Infectious Diseases 197: 1119–1123. 18462161 10.1086/529526

[27] Oleinikov AV, Rossnagle E, Francis S, Mutabingwa TK, Fried M, et al (2007) Effects of sex, parity, and sequence variation on seroreactivity to candidate pregnancy malaria vaccine antigens. J Infect Dis 196: 155–164 Epub 2007 May 2023. 17538896 10.1086/518513

[28] Lineweaver H, Burk D (1934) The Determination of Enzyme Dissociation Constants. Journal of the American Chemical Society 56: 658–666.

[29] Deleage G, Blanchet C, Geourjon C (1997) Protein structure prediction. Implications for the biologist. Biochimie 79: 681–686. 9479451 10.1016/s0300-9084(97)83524-9

[30] Tolia NH, Enemark EJ, Sim BK, Joshua-Tor L (2005) Structural basis for the EBA-175 erythrocyte invasion pathway of the malaria parasite Plasmodium falciparum. Cell 122: 183–193. 16051144 10.1016/j.cell.2005.05.033

[31] Springer AL, Smith LM, Mackay DQ, Nelson SO, Smith JD (2004) Functional interdependence of the DBLbeta domain and c2 region for binding of the Plasmodium falciparum variant antigen to ICAM-1. Mol Biochem Parasitol 137: 55–64. 15279951 10.1016/j.molbiopara.2004.03.019

[32] Bengtsson A, Joergensen L, Rask TS, Olsen RW, Andersen MA, et al (2013) A Novel Domain Cassette Identifies Plasmodium falciparum PfEMP1 Proteins Binding ICAM-1 and Is a Target of Cross-Reactive, Adhesion-Inhibitory Antibodies. J Immunol 190: 240–249. 23209327 10.4049/jimmunol.1202578 PMC3539686

[33] Hughes KR, Biagini GA, Craig AG (2010) Continued cytoadherence of Plasmodium falciparum infected red blood cells after antimalarial treatment. Mol Biochem Parasitol 169: 71–78 Epub 2009 Oct 2001. 19800372 10.1016/j.molbiopara.2009.09.007 PMC2814047

[34] Gray C, McCormick C, Turner G, Craig A (2003) ICAM-1 can play a major role in mediating P. falciparum adhesion to endothelium under flow. Mol Biochem Parasitol 128: 187–193. 12742585 10.1016/s0166-6851(03)00075-6

[35] Gardner JP, Pinches RA, Roberts DJ, Newbold CI (1996) Variant antigens and endothelial receptor adhesion in Plasmodium falciparum. Proc Natl Acad Sci U S A 93: 3503–3508. 8622966 10.1073/pnas.93.8.3503 PMC39639

