# Supplementary material for: Structure-Function-Immunogenicity Studies of PfEMP1 Domain DBL2βPF11_0521, a Malaria Parasite Ligand for ICAM-1
Source: PLoS One. 2013 Apr 12;8(4):e61323. doi: 10.1371/journal.pone.0061323 (PMC3625211; doi:10.1371/journal.pone.0061323)
Supplement: Figure S1 — The nucleotide sequence for full-length DBL2βPF11_0521 domain and corresponding protein sequences for the various truncation and point-mutation constructs in various vectors. (DOCX) [file pone.0061323.s001.docx]

**Supplementary Figure S1**. The nucleotide sequence for full-length DBL2β_PF11_0521_ domain and corresponding protein sequences for the various truncation and point-mutation constructs in various vectors.

Nucleotide sequence and corresponding amino acid sequence of full-length DBL2β_PF11_0521_ domain cloned into pHisAdEx vector [21]. Cloning sites (Bam HI and Eco RI) are underlined.

GGATCCag**AAC**CCGTGTGCTAAACCTCATGGTAAGAAACTTGCGACTGTGAAACAAATCGCACAATATTATAAA**CGC**AAG**GCA**TATATACAATTGAATGAGCGCGGTAGTAGAAGTGCTTTGAAAGGGGATGCGTCACAAGGTCAATATGATCGTGGAGGTAAAGCAGATGACTTCAAGACAAAATTATGTGAAATAAATGAAAAGCATTCGAATGCTCGCAGTAATTCACTAAATCCATGTAATGGAAAAGATAATAATAAAGTAAGGTTTAATGTAGGAACACCATGGCAGAGTGGGGAAAAAATAGCTACAGCAACTGATGTTTATTTACCACCACGACGTCAACATTTTTGTACATCAAACTTGGAATATTTAATTAATGGTGGTCATCAAGCAATTCTGAATGTTAAAAATGGAAAGATTAATCATTCCTTTTTGGGAGATGTACTTCTGGCAGCAAAGTACCAAGCACAACATACAATGAAGGACTATAAATCTAAAAATGACAAGGAAGGTATATGTCGAGCCATACGTTACAGTTTTGCCGATATAGGTGATATAATTAAAGGAACAGATTTATGGGATAAAGATGGTGGAGAGATTAAAACACAAAATCATTTGGTGACAATATTTGATAAAATTAAAGCGCAACTTCCTAAAGACATCAAAGGAAAATATACCGGAACCAAACATTTAGAATTACGTAAAGACTGGTGGGAAGCAAATCGTGATCAAGTGTGGAAGGCGATGCAGTGTGGCAACGACAACCCATGTAGTGGTGAAAGTGATCATACACCGCTACATGACTACATCCCTCAAAGGTTACGCTGGATGACAGAATGGGCCGAATGGTATTGCAAGGAGCAGTCAAGGTTGTATGACAAGTTGAAGGTGTGTGAGGAGTGTATGAGAAAAGGGGAATCATGTACGAAAGGGAGTGGTGAGTGTGCAACGTGCAAGGAAGCATGTGAAGAATATAATAAAGAAATAAAAAAATGGGAACAACAATGGGATGCAATATCATACAAATACCTAATGTTATAC**GCA**AAAGCACGAATTACTGCTATTAATGGTGGTCCTGGGTATTATAATACGGAAGTACAGGAGGAAGACAAACCTGTCGTTGACTTTTTGTACAATTTATATCTACAAAATGGTGGCAAAAAAGGTCCCCCTCCTGACACTCATCGTGTTAAAGCTCTTATCGCACGTGTTAAACGTGATGCCGCACGTAATCGTGTTAAACGTGCTGATGGTAGTAGTGCCACTAGGGTAACCGCCACCACCACGATCACCCCCTACAGCACCGCTGCCGGCTATATTCATCAAGAAGCACATATTGGTGATTGTCAGAAACAAACACAATTTTGTAAAAACAAAAATGGTAGTGACGTTAGTGATACAGAGGCCGATCCCACTTATGCCTTTAGGGATAAACCACATGATCATGATACAGCATGTAAATGCAAGGACAGACAGCCGGAATTAGTAACGGAAAAAAAAAAAGATGATGAAGGCGAAGAACAAGAAGACGAACCACCAAAACCAAAACCTCCTTCTACTCCAAAT**CCG**gGAATTC

COS7 expressed protein consists of:

22 amino acid residues resulted from the vector and cloning site translation —**N**PCAKPHGKKLATVKQIAQYYK**R**K**A**YIQLNERGSRSALKGDASQGQYDRGGKADDFKTKLCEINEKHSNARSNSLNPCNGKDNNKVRFNVGTPWQSGEKIATATDVYLPPRRQHFCTSNLEYLINGGHQAILNVKNGKINHSFLGDVLLAAKYQAQHTMKDYKSKNDKEGICRAIRYSFADIGDIIKGTDLWDKDGGEIKTQNHLVTIFDKIKAQLPKDIKGKYTGTKHLELRKDWWEANRDQVWKAMQCGNDNPCSGESDHTPLHDYIPQRLRWMTEWAEWYCKEQSRLYDKLKVCEECMRKGESCTKGSGECATCKEACEEYNKEIKKWEQQWDAISYKYLMLY**A**KARITAINGGPGYYNTEVQEEDKPVVDFLYNLYLQNGGKKGPPPDTHRVKALIARVKRDAARNRVKRADGSSATRVTATTTITPYSTAAGYIHQEAHIGDCQKQTQFCKNKNGSDVSDTEADPTYAFRDKPHDHDTACKCKDRQPELVTEKKKDDEGEEQEDEPPKPKPPSTPN**P** — Trans-membrane domain and cytoplasmic tail followed by GFP sequence

First and last amino acid residues of the domain and corresponding codons are shown in **blue bold font**. Number of AA residues in DBL2β_PF11_0521_ domain is 522 (1 through 522). Mutated amino acid residues and corresponding codons are indicated by **red bold font**. Mutations made: A25K; R23A + A25K; A347L; A347H; A347Y. All mutants were expressed in COS-7 cells, proteins were immobilized on BioPlex beads and studied for ICAM-1 binding.

Below are shown AA sequences of all DBL2β_PF11_0521_ domain fragments expressed in *E. coli*.

Cloning into pET28b for expression in *E. coli* was performed using NcoI and XhoI restriction digestion sites. All resulting protein products have the structure:

MG-Domain fragment-LEHHHHHH (amino acid residues in one-letter code)

Full-length domain (1-520)

MG**N**PCAKPHGKKLATVKQIAQYYKRKAYIQLNERGSRSALKGDASQGQYDRGGKADDFKTKLCEINEKHSNARSNSLNPCNGKDNNKVRFNVGTPWQSGEKIATATDVYLPPRRQHFCTSNLEYLINGGHQAILNVKNGKINHSFLGDVLLAAKYQAQHTMKDYKSKNDKEGICRAIRYSFADIGDIIKGTDLWDKDGGEIKTQNHLVTIFDKIKAQLPKDIKGKYTGTKHLELRKDWWEANRDQVWKAMQCGNDNPCSGESDHTPLHDYIPQRLRWMTEWAEWYCKEQSRLYDKLKVCEECMRKGESCTKGSGECATCKEACEEYNKEIKKWEQQWDAISYKYLMLYAKARITAINGGPGYYNTEVQEEDKPVVDFLYNLYLQNGGKKGPPPDTHRVKALIARVKRDAARNRVKRADGSSATRVTATTTITPYSTAAGYIHQEAHIGDCQKQTQFCKNKNGSDVSDTEADPTYAFRDKPHDHDTACKCKDRQPELVTEKKKDDEGEEQEDEPPKPKPPSTPLEHHHHHH

Short domain (91-391)

MGGTPWQSGEKIATATDVYLPPRRQHFCTSNLEYLINGGHQAILNVKNGKINHSFLGDVLLAAKYQAQHTMKDYKSKNDKEGICRAIRYSFADIGDIIKGTDLWDKDGGEIKTQNHLVTIFDKIKAQLPKDIKGKYTGTKHLELRKDWWEANRDQVWKAMQCGNDNPCSGESDHTPLHDYIPQRLRWMTEWAEWYCKEQSRLYDKLKVCEECMRKGESCTKGSGECATCKEACEEYNKEIKKWEQQWDAISYKYLMLYAKARITAINGGPGYYNTEVQEEDKPVVDFLYNLYLQNGGKKGPPPLEHHHHHH

3-Helix bundle (267-391)

MGDYIPQRLRWMTEWAEWYCKEQSRLYDKLKVCEECMRKGESCTKGSGECATCKEACEEYNKEIKKWEQQWDAISYKYLMLYAKARITAINGGPGYYNTEVQEEDKPVVDFLYNLYLQNGGKKGPPPLEHHHHHH

NTF (1-69)

MGNPCAKPHGKKLATVKQIAQYYKRKAYIQLNERGSRSALKGDASQGQYDRGGKADDFKTKLCEINEKHSNLEHHHHHH
