# Supplementary material for: Structure-Function-Immunogenicity Studies of PfEMP1 Domain DBL2βPF11_0521, a Malaria Parasite Ligand for ICAM-1
Source: PLoS One. 2013 Apr 12;8(4):e61323. doi: 10.1371/journal.pone.0061323 (PMC3625211; doi:10.1371/journal.pone.0061323)
Supplement: Figure S2 — Structural elements of interacting ICAM-1 and DBLβ in the model of Bertonati and Tramontano. Model [24] was visualized (A) and amino acid substitution in loop 4 (B) was modeled using Deep View Swiss-PDB viewer. In panel A loops 1 through 4 participating in interactions of DBLβ domain with ICAM-1 are shown in red, the rest of the molecule in green, ICAM-1 molecule in yellow. Ala286 residue in loop 4 that is involved in contacts with ICAM-1 is shown in blue. Y motif is circled. In panel B amino acid residue 286 (Ala or Tyr) is shown in pink. Amino acid residues Asn (N) and Tyr (Y), which are located at the beginning and the end of the loop 4, are indicated. Residues in the vicinity of the position 286 are shown in space-filling shape with CPK coloring. Substitution Ala286Tyr creates an additional hydrogen bond (arrowhead in panel C) between Tyr286 and Arg113 (numbers according to [24]). (PPTX) [file pone.0061323.s002.pptx]

## Slide 1
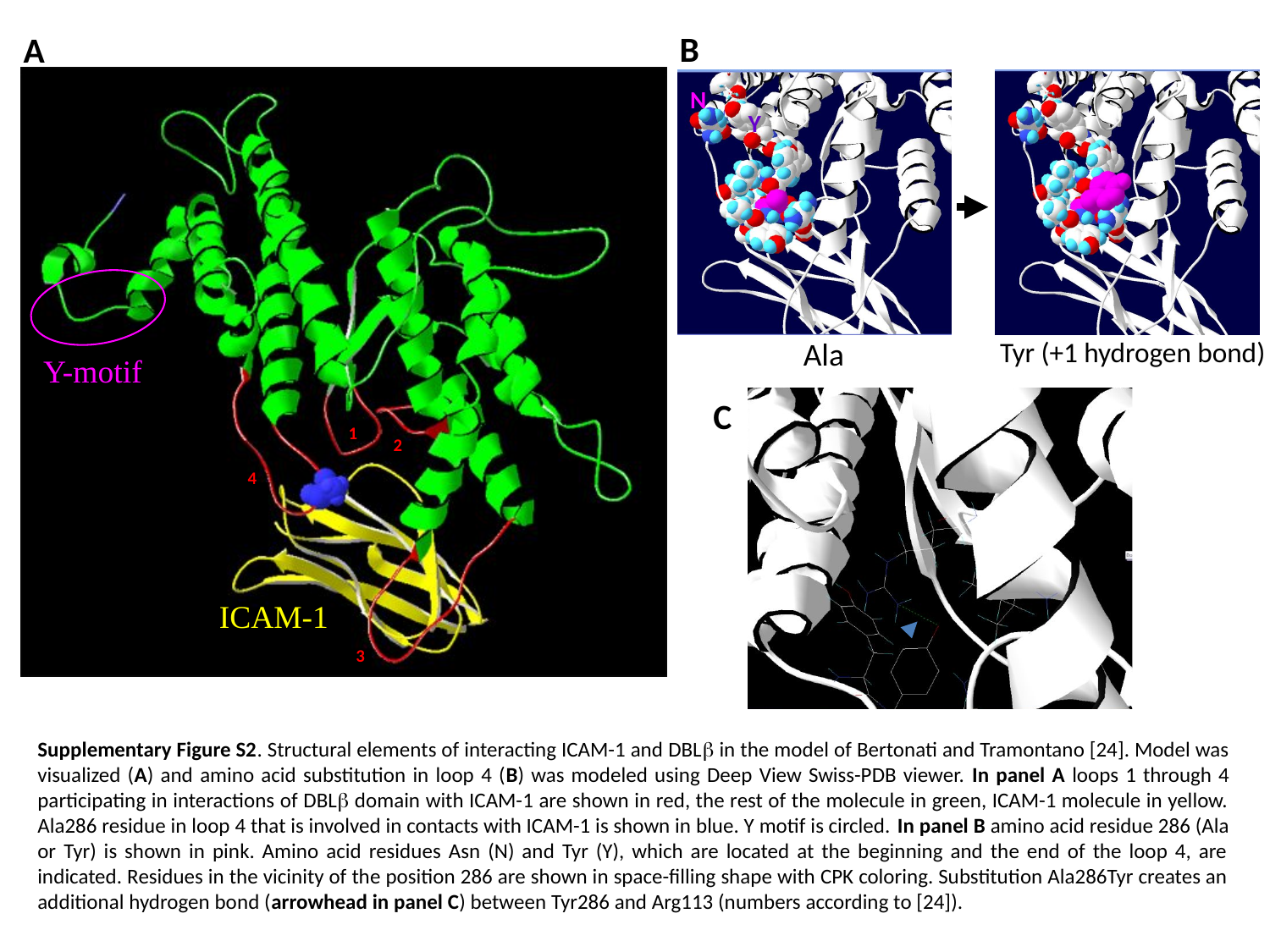

B
A
1
2
4
3
N
Y
Tyr (+1 hydrogen bond)
Ala
Y-motif
C
ICAM-1
Supplementary Figure S2. Structural elements of interacting ICAM-1 and DBLb in the model of Bertonati and Tramontano [24]. Model was visualized (A) and amino acid substitution in loop 4 (B) was modeled using Deep View Swiss-PDB viewer. In panel A loops 1 through 4 participating in interactions of DBLb domain with ICAM-1 are shown in red, the rest of the molecule in green, ICAM-1 molecule in yellow. Ala286 residue in loop 4 that is involved in contacts with ICAM-1 is shown in blue. Y motif is circled. In panel B amino acid residue 286 (Ala or Tyr) is shown in pink. Amino acid residues Asn (N) and Tyr (Y), which are located at the beginning and the end of the loop 4, are indicated. Residues in the vicinity of the position 286 are shown in space-filling shape with CPK coloring. Substitution Ala286Tyr creates an additional hydrogen bond (arrowhead in panel C) between Tyr286 and Arg113 (numbers according to [24]).
