# Supplementary material for: Structure-Function-Immunogenicity Studies of PfEMP1 Domain DBL2βPF11_0521, a Malaria Parasite Ligand for ICAM-1
Source: PLoS One. 2013 Apr 12;8(4):e61323. doi: 10.1371/journal.pone.0061323 (PMC3625211; doi:10.1371/journal.pone.0061323)
Supplement: Figure S3 — Predicted secondary structure of N-terminal sub-domain and amino acid residue mutations in the first α-helix of DBL2βPF11_0521. ICAM-1 binding and non-binding domains [20], [21] are grouped. Color of amino acid residues: purple – conserved at least in ICAM-1 binding sequences; blue – semi-conserved; red – having significantly different physical-chemical character from the majority of amino acid residues in this position that may affect structure or/and function of the domain. Site-directed mutations: R23→A, A25→K. (PPTX) [file pone.0061323.s003.pptx]

## Slide 1
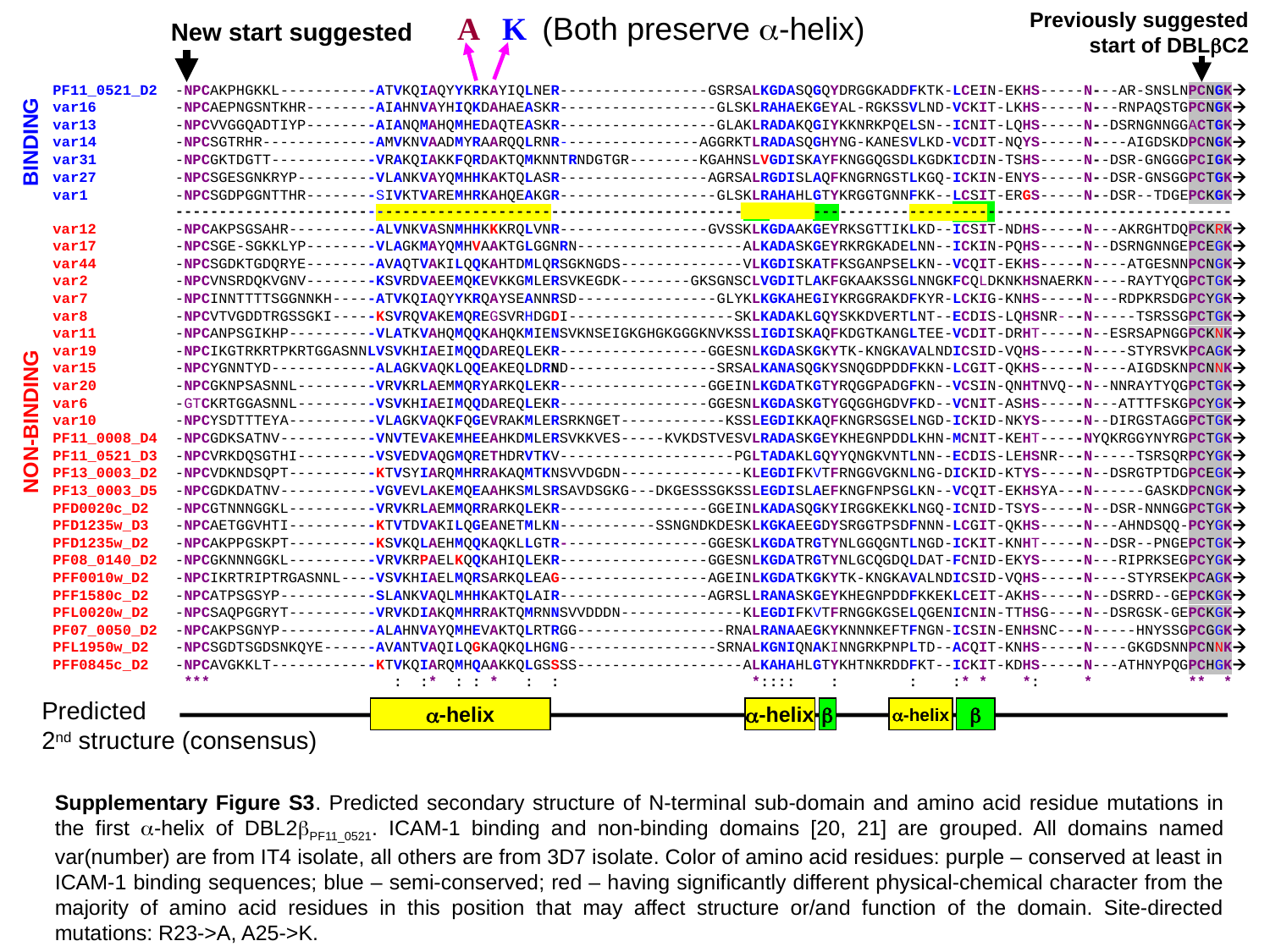

Previously suggested start of DBLbC2
A
K
(Both preserve a-helix)
New start suggested
BINDING
NON-BINDING
Predicted
2nd structure (consensus)
a-helix
a-helix
b
a-helix
b
Supplementary Figure S3. Predicted secondary structure of N-terminal sub-domain and amino acid residue mutations in the first a-helix of DBL2bPF11_0521. ICAM-1 binding and non-binding domains [20, 21] are grouped. All domains named var(number) are from IT4 isolate, all others are from 3D7 isolate. Color of amino acid residues: purple – conserved at least in ICAM-1 binding sequences; blue – semi-conserved; red – having significantly different physical-chemical character from the majority of amino acid residues in this position that may affect structure or/and function of the domain. Site-directed mutations: R23->A, A25->K.
