# Supplementary material for: Structure-Function-Immunogenicity Studies of PfEMP1 Domain DBL2βPF11_0521, a Malaria Parasite Ligand for ICAM-1
Source: PLoS One. 2013 Apr 12;8(4):e61323. doi: 10.1371/journal.pone.0061323 (PMC3625211; doi:10.1371/journal.pone.0061323)
Supplement: Figure S4 — Determination of equilibrium dissociation constant KD for ICAM-1 binding to PF11_0521 DBL2 domain. Kinetics of binding for both E. coli expressed/refolded (ECO, N = 18) and COS-7 expressed (COS, N = 14) domains was measured in 4 independent experiments. 95% confidence intervals (CI) shown by dashed lines. Error bars are standard errors of mean. N, number of replicates for each concentration point. Formulas show linear regressions. V, initial velocity of binding; C, concentration of ICAM-1; min, minutes, AU, arbitrary units. (PPTX) [file pone.0061323.s004.pptx]

## Slide 1
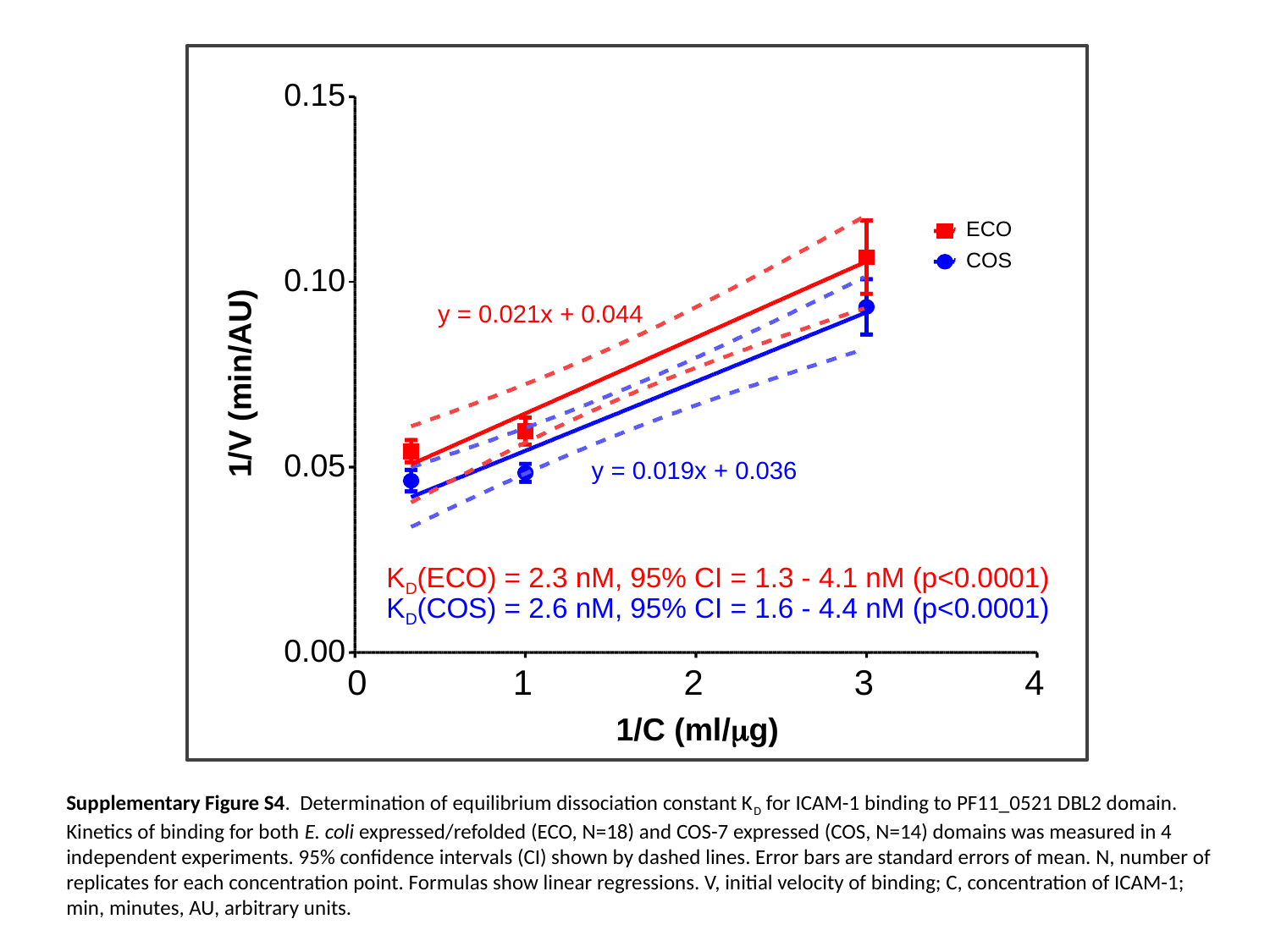

0.15
ECO
COS
0.10
y = 0.021x + 0.044
1/V (min/AU)
0.05
y = 0.019x + 0.036
KD(ECO) = 2.3 nM, 95% CI = 1.3 - 4.1 nM (p<0.0001)
KD(COS) = 2.6 nM, 95% CI = 1.6 - 4.4 nM (p<0.0001)
0.00
0
1
2
3
4
1/C (ml/mg)
Supplementary Figure S4. Determination of equilibrium dissociation constant KD for ICAM-1 binding to PF11_0521 DBL2 domain. Kinetics of binding for both E. coli expressed/refolded (ECO, N=18) and COS-7 expressed (COS, N=14) domains was measured in 4 independent experiments. 95% confidence intervals (CI) shown by dashed lines. Error bars are standard errors of mean. N, number of replicates for each concentration point. Formulas show linear regressions. V, initial velocity of binding; C, concentration of ICAM-1; min, minutes, AU, arbitrary units.
