# Supplementary material for: Structure-Function-Immunogenicity Studies of PfEMP1 Domain DBL2βPF11_0521, a Malaria Parasite Ligand for ICAM-1
Source: PLoS One. 2013 Apr 12;8(4):e61323. doi: 10.1371/journal.pone.0061323 (PMC3625211; doi:10.1371/journal.pone.0061323)
Supplement: Table S1 — Summary of results of binding and inhibition of binding of ICAM-1 receptor obtained with various constructs of DBLβPF11_0521 and antibodies against these constructs. (DOCX) [file pone.0061323.s005.docx]

Supplementary Table S1. Summary of results of binding and inhibition of binding of ICAM-1 receptor obtained with various constructs of DBLβ_PF11_0521_ and antibodies against these constructs

Domain ICAM-1 binding antibodies* Reactivity with protein Inhibition of ICAM-1 binding

variant* to F(C) domain F(C) F(E) Sh 3H NTF to Full-length (COS-7) domain

Full-length Yes Yes* + + - - + Yes

(DNA or COS-7)*

Full-length Yes Yes* + + + + + Yes

(*E. coli*)*

Short No Yes + + + + - No

(*E. coli*)

3-helix No Yes + + + + - No

(*E. coli*)

NTF No Yes + + - - + No

(*E. coli*)

Notes: *, Full-length domain variant was expressed in COS-7 cells and in *E. coli*, antibodies were raised to full-length domain either by DNA immunization or by immunization with E. coli expressed domain; F(C), Full-length domain expressed in COS-7 cells; F(E), Full-length domain expressed in *E. coli*; Sh, Short construct; 3H, 3-helix construct; NTF, N-terminal fragment. ” +“ and “-“ indicate detectable reactivity and absence of reactivity.
